# Adult Mosquitoes Infected with Bacteria Early in Life Have Stronger Antimicrobial Responses and More Hemocytes after Reinfection Later in Life

**DOI:** 10.3390/insects11060331

**Published:** 2020-05-28

**Authors:** Joseph C. Powers, Raymar Turangan, Bryan A. Joosse, Julián F. Hillyer

**Affiliations:** Department of Biological Sciences, Vanderbilt University, Nashville, TN 37235, USA; joseph.c.powers@vanderbilt.edu (J.C.P.); raymar.turangan@vanderbilt.edu (R.T.); bryan.a.joosse@vanderbilt.edu (B.A.J.)

**Keywords:** *Anopheles gambiae*, immunity, insect, nitric oxide synthase, phagocytosis, prophenoloxidase, survival

## Abstract

The immunological strategies employed by insects to overcome infection vary with the type of infection and may change with experience. We investigated how a bacterial infection in the hemocoel of the African malaria mosquito, *Anopheles gambiae*, prepares the immune system to face a subsequent bacterial infection. For this, adult female mosquitoes were separated into three groups—unmanipulated, injured, or infected with *Escherichia coli*—and five days later all the mosquitoes were infected with a different strain of *E. coli*. We found that an injury or a bacterial infection early in life enhances the ability of mosquitoes to kill bacteria later in life. This protection results in higher mosquito survival and is associated with an increased hemocyte density, altered phagocytic activity by individual hemocytes, and the increased expression of nitric oxide synthase and perhaps prophenoloxidase 6. Protection from a second infection likely occurs because of heightened immune awareness due to an already existing infection instead of memory arising from an earlier, cured infection. This study highlights the dynamic nature of the mosquito immune response and how one infection prepares mosquitoes to survive a subsequent infection.

## 1. Introduction

The B and T cells of vertebrate animals collectively display a vast arsenal of receptors, each of which recognizes distinct molecular features found in pathogens [1]. Together with the ability of these cells to expand clonally and produce memory cells, vertebrate animals can quickly and efficiently respond to a specific pathogen during a secondary encounter. Insects lack B and T cells but have evolved mechanisms to protect themselves from reinfection [2,3,4,5,6]. This protection can be due to a sustained immune response, a recall response, or an immune shift, and can take place either with or without the resolution of the initial infection. Protection sometimes is specific to the pathogen already encountered, but most often protection is conferred against other types of pathogens as well [7,8,9,10,11]. Although this kind of protection—often referred to as immune priming—occurs in multiple insect orders, it does not protect against all types of infection and it does not occur in all insects [12,13,14,15].

In mosquitoes (order Diptera, family Culicidae) from both major subfamilies (Anophelinae and Culicinae), an infection can confer protection from a subsequent infection. Infection with bacteria, microsporidian parasites, or malaria parasites protects mosquitoes from a secondary infection with malaria parasites and filarial worms [16,17,18,19,20,21,22,23], and a *Wolbachia* infection protects mosquitoes from infection with the dengue and Zika viruses [24,25]. Some of this protection is conferred while the primary infection ensues [16,19], but mosquitoes cured of *Plasmodium berghei* or exposed to the inactivated dengue virus remain protected from reinfection with that same pathogen [17,18,20,26]. However, there is significant variability in how an immune challenge protects from an infection later in life. *Asaia* sp., for example, confers protection against *P. berghei* in *Anopheles stephensi* but not in *Anopheles gambiae*, and only some bacterial infections confer protection from a subsequent bacterial infection in *Aedes aegypti* [27,28]. Likewise, although *Wolbachia* protects *A. aegypti* from the dengue and Zika viruses, it increases *Culex tarsalis* susceptibility to the West Nile virus [24,25,29]. These *Wolbachia*-related differences appear to depend on the combination of bacterial strain and mosquito species; for example, some combinations render mosquitoes more resistant to malaria, whereas others enhance susceptibility [30,31,32,33].

In the present study, we queried whether a bacterial infection in the African malaria mosquito, *A. gambiae*, activates the immune system in a way that grants protection from a subsequent bacterial infection. We uncovered that an *Escherichia coli* infection in the hemocoel early in life increases the ability of mosquitoes to kill *E. coli* that reinfect them later in life. This heightened antibacterial response upon a second infection is accompanied by a surge in hemocytes and nitric oxide synthase expression and increases the probability that mosquitoes survive infection.

## 2. Materials and Methods

### 2.1. Mosquito Rearing and Maintenance

*A. gambiae* Giles sensu stricto (G3 strain) were reared and maintained in an environmental chamber at 27 °C and 75% relative humidity under a 12 hr:12 hr light/dark photoperiod [34]. Adult mosquitoes were fed a 10% sucrose solution ad libitum.

### 2.2. General Experimental Design

**Figure 1:**
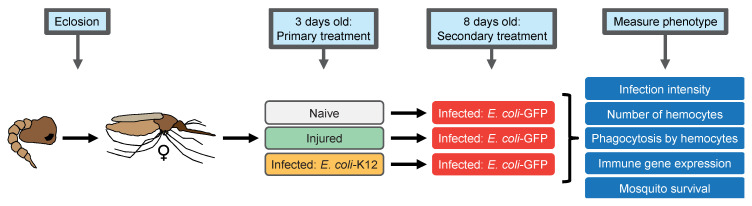
Diagrammatic representations of the experimental approach. At 3 days after eclosion, mosquitoes were subjected to one of three primary treatments: naïve (unmanipulated), injured, or infected with *E. coli*-K12. At day 8, all the mosquitoes were infected with *E. coli*-GFP and several phenotypes were measured. Infection intensity was measured at 1, 3, and 7 days after the *E. coli*-GFP infection. The number of hemocytes and immune gene expression were assayed 1 day after the *E. coli*-GFP infection. Phagocytosis was measured 1 hr after the *E. coli*-GFP infection, and survival was tracked every day after the *E. coli*-GFP infection until all the mosquitoes had died.

Three days after eclosion, female adult mosquitoes were either left unmanipulated (naïve), were injured, or were infected with *E. coli*-K12—this is the primary treatment. Five days later (8 days of adulthood), every mosquito was infected with *E. coli*-GFP—this is the secondary treatment. Then, several phenotypes were assayed (Figure 1).

### 2.3. Intravital Injections and Infections

The mosquitoes were anesthetized by placing them in a −20 °C environment for 30–40 sec and then transferred to a Petri dish placed on top of ice. For both primary and secondary treatments, a finely pulled capillary glass needle was inserted into the hemocoel through the thoracic anepisternal cleft, and 69 nL of a solution were injected under the control of a Nanoject II Auto-Nanoliter Injector (Drummond Scientific Company, Broomall, PA, USA). The mosquitoes were then returned to 27 °C. The mosquitoes were injected with either sterile Luria–Bertani’s rich nutrient medium (LB broth; injury) or with bacteria in LB broth (infection). Other mosquitoes were anesthetized but otherwise left unmanipulated (naïve).

Bacterial infections were conducted using two strains of *E. coli*: a parental K12 strain (*E. coli*-K12) and a derived DH5 alpha strain that was tetracycline-resistant and expressed GFP (*E. coli*-GFP). Both strains were grown overnight in LB broth at 37 °C in a shaking incubator, the absorbance of each culture was measured using a Biophotometer Plus spectrophotometer (Eppendorf, Hamburg, Germany) and normalized to OD_600_ = 2, and the culture was injected into the hemocoel.

### 2.4. Bacterial Infection Intensity

At 1, 3, or 7 days after the *E. coli*-GFP infection (the secondary treatment), the mosquitoes were individually homogenized in sterile water. Each homogenate was diluted and spread on an LB agar plate containing tetracycline. The plates were incubated at 37 °C and the colony forming units (CFUs) were counted 18 h later. Using the number of CFUs and the dilution factor, the absolute number of bacteria present in the hemocoel of each mosquito at the time of homogenization—the infection intensity—was calculated [35,36]. Because only *E. coli*-GFP—and not *E. coli*-K12—were resistant to tetracycline, only bacteria originating from the secondary treatment grew on the LB agar plates. Four independent trials were conducted for days 1 and 3, and three independent trials were conducted for day 7. For each day, between 45 and 64 mosquitoes were analyzed per primary/secondary treatment combination and the data from all the trials were aggregated. The data were separated by primary treatment and the number of days post-secondary treatment and tested for normality using the Shapiro–Wilk test. Because not all groups conformed to a normal distribution, the data were analyzed using the Kruskal–Wallis test followed by Dunn’s post-hoc test. For every experiment in this study, the differences were deemed significant at *p* < 0.05.

### 2.5. Quantification of the Number of Circulating Hemocytes Per Mosquito

At 1 day after secondary treatment, the circulating hemocytes were collected by volume displacement, also known as perfusion [35,37]. Specifically, an incision was made in the last abdominal segment, and the mosquito was then held vertically on a vacuum saddle with the abdomen pointing down. A capillary glass needle was inserted through the cervical (neck) membrane, Grace’s insect medium was injected into the hemocoel, and the first 5 drops of diluted hemolymph that exited the body at the last abdominal segment were collected on a 1 cm diameter etched ring on a Rite-On glass slide (Gold Seal; Portsmouth, NH, USA). The slide was placed in a humidity chamber for 20 min to allow the hemocytes to adhere to the glass, and the hemocytes were then fixed and stained using Hema 3 (Fisher Scientific, Pittsburgh, PA, USA), dried, and mounted under a coverslip using Poly-Mount (Polysciences, Warrington, PA, USA) [36]. The hemocytes were viewed under differential interference contrast (DIC) illumination on a Nikon 90i light microscope (Nikon Corp., Tokyo, Japan), and the total number of circulating hemocytes in each mosquito was counted. Three biological trials were conducted, each composed of 10 mosquitoes per primary/secondary treatment combination. The data from all the trials were aggregated, and because not all groups conformed to a normal distribution (Shapiro–Wilk), the data were analyzed using a Kruskal–Wallis test followed by Dunn’s post-hoc test.

### 2.6. Quantification of Phagocytosis by Circulating Hemocytes

At 1 hr after secondary treatment, a time when the phagocytosis response has been activated but the infection has not been controlled [35,36], hemolymph was collected by perfusion. Hemocytes were allowed to adhere to the slide for 20 min, and the cells were fixed and stained for 15 min by adding 4% paraformaldehyde (Electron Microscopy Sciences, Hatfield, PA, USA) and Hoechst 33342 nuclear stain (Invitrogen, Carlsbad, CA, USA) in phosphate buffered saline (PBS). Slides were then mounted with coverslips using Aqua-Poly/Mount (Polysciences) and viewed under simultaneous DIC and fluorescence illumination on the Nikon 90i microscope [37]. For each mosquito, the number of GFP-*E. coli* that had been phagocytosed by each of the first 50 hemocytes viewed was counted. From these counts, the phagocytic index—defined as the percentage of hemocytes that phagocytosed *E. coli*-GFP—and the phagocytic capacity—defined as the number of *E. coli*-GFP per hemocyte or per phagocytic hemocyte—were calculated [35,36,37]. It is important to note that *E.coli*-K12 are not fluorescent, so they were not counted in this assay. Three biological trials were conducted, each composed of between 8 and 11 mosquitoes per primary/secondary treatment combination, and the data were aggregated. Data on the phagocytic index did not conform to a normal distribution (Shapiro–Wilk), and were analyzed using a Kruskal–Wallis test followed by Dunn’s post-hoc test. Data on the phagocytic capacity conformed to a normal distribution (Shapiro–Wilk), and were analyzed by a one-way ANOVA followed by Tukey’s post-hoc test.

### 2.7. Quantification of Gene Expression by Real-Time PCR (RT-PCR)

At 1 day after secondary treatment, RNA was extracted from the mosquito whole bodies. For this, 10–12 mosquitoes were pooled and homogenized in TRIzol reagent (Invitrogen), the RNA was purified, and the RNA was then re-purified using the RNeasy Mini Kit (Qiagen, Valencia, CA, USA). The RNA was treated with DNAse (Promega, Madison, WI) to eliminate any residual genomic DNA contamination, ethanol precipitated, and resuspended in water. Then, 1–5 μg of RNA was used for a complementary DNA (cDNA) synthesis, which was performed using an oligo dT primer and Invitrogen’s SuperScript III First-Strand Synthesis System for RT-PCR [34,35].

The RT-PCR was performed using cDNA as a template, gene specific primers, and Power SYBR Green PCR Master Mix (Applied Biosystems, Foster City, CA, USA) on a Bio-Rad CFX Connect Real-Time PCR Detection System (Hercules, CA, USA). The thermal cycle conditions were 50 °C for 2 min, 95 °C for 10 min, and 40 cycles each composed of 95 °C for 15 sec and 60 °C for 1 min. After the RT-PCR, a melting curve analysis was performed to confirm that only the target sequence was amplified and that there was no genomic DNA contamination. Relative quantification was performed using the 2^−ΔΔC^_T_ method [38], using ribosomal protein S7 (*RPS7*) as the reference and ribosomal protein S17 (*RPS17*) as a control [34,35]. Four biological trials were conducted, and each was analyzed in duplicate. The two technical replicates of a trial were averaged and this value used as the outcome of the trial. Because the small sample size (n = 4) precluded the accurate testing of normality, the data were analyzed using a Kruskal–Wallis test. A list of the genes assayed in this study, including their VectorBase IDs and the primers used, is presented in Table 1.

### 2.8. Mosquito Survival

After secondary treatment, the survival of the mosquitoes in each of the three primary/secondary treatment combinations was tracked until all the mosquitoes had died. Data from 4 independent trials were aggregated and the mosquito survival was compared using the log-rank (Mantel–Cox) test. The survival of 483, 343, and 350 mosquitoes was tracked for the naïve, injured, and *E. coli*-K12 primary treatment groups, respectively.

## 3. Results

### 3.1. A Bacterial Infection in the Hemocoel Early in Life Increases the Ability of Mosquitoes to Kill Bacteria Acquired Later in Life

**Figure 2:**
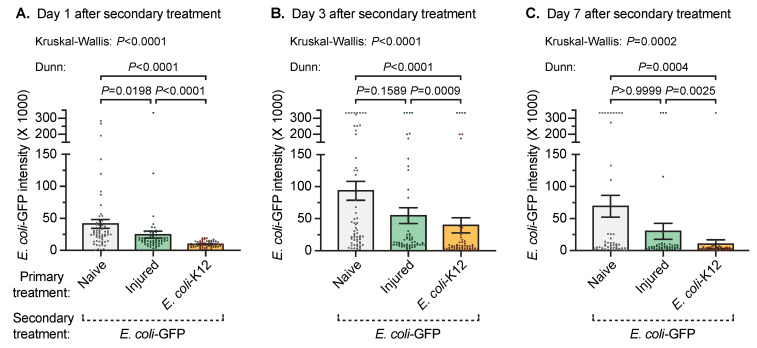
Infection intensity after secondary treatment. Five days after the primary treatment of naïve, injury, or infection with *E. coli*-K12, all the mosquitoes were infected with *E. coli*-GFP and the number of *E. coli*-GFP in their hemocoel was quantified 1 (**A**), 3 (**B**), or 7 (**C**) days later. Column heights mark the mean and whiskers denote the standard error of the mean (SEM). Circles mark the infection intensity of individual mosquitoes.

To begin to determine whether an infection acquired early in adulthood alters the immune response against an infection acquired later in adulthood, 3-day-old mosquitoes were either left unmanipulated (naïve), were injured, or were infected with *E. coli*-K12. Then, at 8 days of age, all the mosquitoes were infected with *E. coli*-GFP, and the *E. coli*-GFP infection intensity was measured 1, 3, or 7 days later (Figure 1 and Figure 2).

At 1 day after the *E. coli*-GFP secondary treatment, the intensity of infection in the mosquitoes that received the naïve primary treatment was 1.7 times higher than in mosquitoes that received the injury primary treatment, and 4.4 times higher than in mosquitoes that received the *E. coli*-K12 infection primary treatment. The same trend was observed at 3 and 7 days after *E. coli*-GFP infection. Specifically, the mosquitoes that received the naïve primary treatment had 1.7 and 2.4 times more *E. coli*-GFP in their hemocoel 3 days after *E. coli*-GFP infection than mosquitoes that received the injury or *E. coli*-K12 primary treatment, respectively. At day 7 after the secondary treatment, the *E. coli*-GFP infection intensity in mosquitoes that received the naïve primary treatment was 2.3 and 6.9 times higher than in mosquitoes that had been injured or infected with *E. coli*-K12 earlier in life, respectively. Therefore, a prior hemocoelic infection, and to a lesser extent a prior injury, enhances the ability of mosquitoes to kill bacteria that infect their hemocoel later in life.

### 3.2. A Prior Infection Augments the Infection-Induced Increase in the Number of Circulating Hemocytes

**Figure 3:**
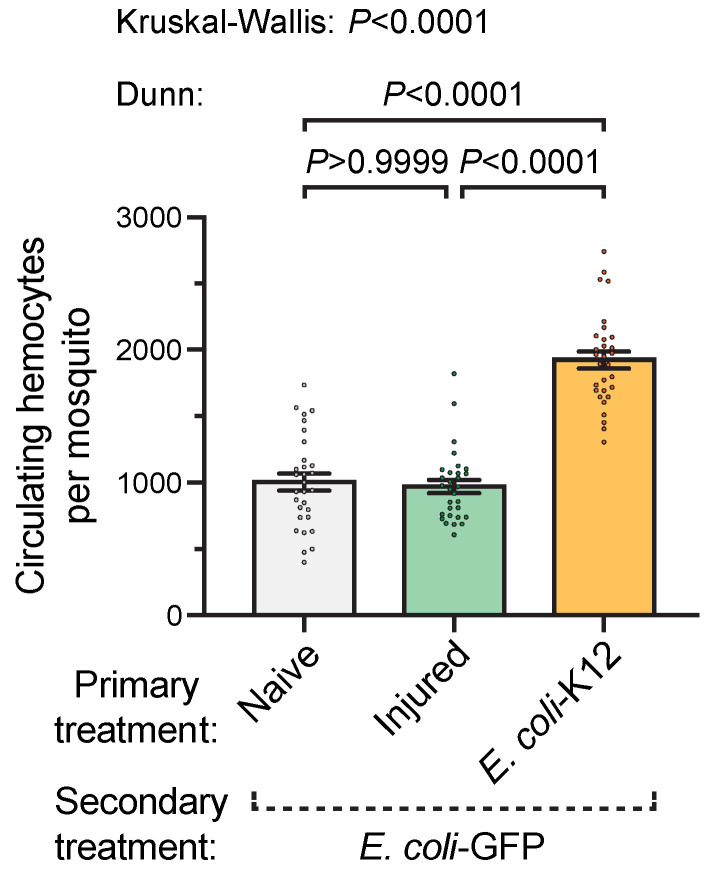
Number of circulating hemocytes after secondary treatment. Five days after the primary treatment of naïve, injury, or infection with *E. coli*-K12, all the mosquitoes were infected with *E. coli*-GFP and the number of circulating hemocytes was counted 1 day later. Column heights mark the mean and whiskers denote the SEM. Circles mark the number of hemocytes in individual mosquitoes.

We then queried whether the increased ability to kill bacteria in the hemocoel is due to increased activity by their primary immune cells—the hemocytes. We began by investigating whether a prior infection alters the number of circulating hemocytes available to fight a secondary infection (Figure 3). At 1 day after the *E. coli*-GFP infection, the mosquitoes from the naïve primary treatment group had an average of 1003 hemocytes. This number was similar in mosquitoes from the injury primary treatment group. However, when mosquitoes were infected with *E. coli*-K12 on day 3 and then with *E. coli*-GFP on day 8, the number of circulating hemocytes on day 9 was 1925. Therefore, when the immune system is stimulated twice relative to when it is stimulated only once, the number of circulating hemocytes almost doubles.

### 3.3. A Prior Infection Decreases the Phagocytic Activity of Individual Hemocytes Against an Infection Acquired Later in Life

**Figure 4:**
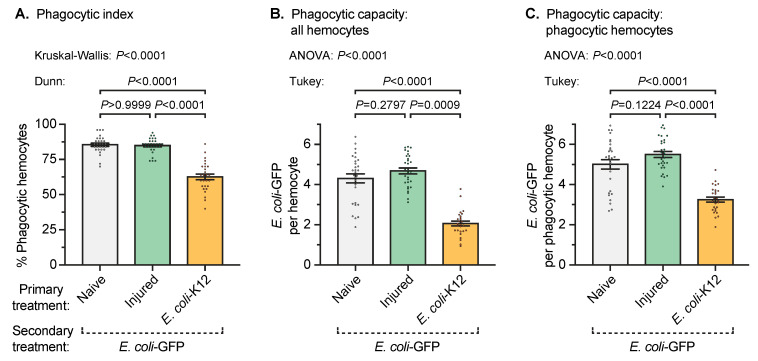
Phagocytic index and phagocytic capacity after secondary treatment. Five days after the primary treatment of naïve, injury, or infection with *E. coli*-K12, all the mosquitoes were infected with *E. coli*-GFP and 1 hr later the following phenotypes were measured: the percentage of hemocytes that phagocytosed *E. coli*-GFP (**A**), the average number of *E. coli*-GFP phagocytosed by each hemocyte (**B**), and the average number of *E. coli*-GFP phagocytosed by each phagocytic hemocyte (**C**). Column heights mark the mean and whiskers denote the SEM. Circles mark the phagocytic activity in individual mosquitoes.

Having established that a prior infection increases the number of circulating hemocytes, we asked whether these hemocytes were more immunologically active. We began by measuring the percentage of hemocytes that phagocytosed *E. coli*-GFP (Figure 4a). In mosquitoes that received the naïve primary treatment, 86% of the hemocytes had phagocytosed at least one *E. coli*-GFP by 1 hr after secondary treatment. This percentage was similar to mosquitoes that had been injured on day 3 and then infected with *E. coli*-GFP on day 8. However, when the mosquitoes received the *E. coli*-K12 primary treatment, the percentage of hemocytes that had phagocytosed at least one *E. coli*-GFP at 1 hr after secondary treatment was reduced to 63%. Therefore, an infection acquired earlier in life decreases the percentage of hemocytes that phagocytose bacteria acquired later in life.

We then quantified the number of bacteria that had been phagocytosed by individual hemocytes (Figure 4b). In mosquitoes that received the naïve primary treatment, the hemocytes had phagocytosed an average of 4.3 *E. coli*-GFP by 1 hr after secondary treatment. This was similar to the phagocytic capacity of the hemocytes from mosquitoes in the injury primary treatment group. However, when the mosquitoes were infected with *E. coli*-K12 on day 3 and then with *E. coli*-GFP on day 8, the number of *E. coli*-GFP per hemocyte was reduced by more than half to an average of 2.1. When only the hemocytes that had phagocytosed bacteria were included in the analysis (discarding hemocytes that had not internalized bacteria), the results were largely the same (Figure 4c). Phagocytic hemocytes from mosquitoes that received the naïve treatment on day 3 and were infected with *E. coli*-GFP on day 8 internalized a similar number of *E. coli*-GFP as phagocytic hemocytes from mosquitoes that were injured on day 3 and were infected with *E. coli*-GFP on day 8. An *E. coli*-K12 infection on day 3, however, decreased the number of *E. coli*-GFP in phagocytic hemocytes by 35% relative to mosquitoes that received the naïve treatment on day 3. Therefore, receiving an infection early in life decreases the phagocytic activity of individual hemocytes against an infection acquired later in life.

### 3.4. An Infection Early in Life Increases the Expression of Nitric Oxide Synthase When Mosquitoes Acquire Another Infection Later in Life

**Figure 5:**
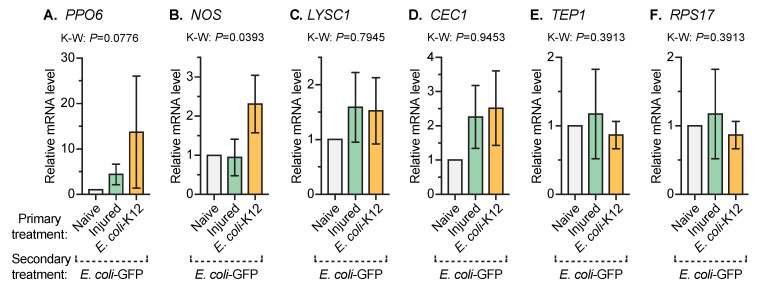
Immune gene expression after secondary treatment. Five days after the primary treatment of naïve, injury, or infection with *E. coli*-K12, all the mosquitoes were infected with *E. coli*-GFP and 1 day later the relative expressions of *PPO6* (**A**), *NOS* (**B**), *LYSC1* (**C**), *CEC1* (**D**), *TEP1* (**E**), and *RPS17* (**F**) were measured by RT-PCR using *RPS7* as the reference. Column heights mark the mean and whiskers denote the SEM.

We then sought to determine whether changes in the expression of some humoral immune genes explain how an infection early in life enhances the ability of mosquitoes to kill bacteria acquired later in life (Figure 5). We focused on five of the best studied immune genes in mosquitoes and one control gene. The largest difference in relative mRNA level observed—although this difference was not statistically significant—was for the melanization-driving prophenoloxidase gene, *PPO6*; mosquitoes that had been infected with *E. coli*-K12 on day 3 and *E. coli*-GFP on day 8 had 14 times more *PPO6* mRNA than mosquitoes that received the naïve treatment on day 3 and were infected with *E. coli*-GFP on day 8. A smaller difference in the relative mRNA level—although statistically significant—was detected for the gene encoding nitric oxide synthase (*NOS*); mosquitoes infected twice had 2.3 times more *NOS* mRNA than mosquitoes that had only been infected later in life. The type of primary treatment did not differentially regulate lysozyme C1 (*LYSC1*), cecropin 1 (*CEC1;* syn. cecropin A or *CECA*), or thioester-containing protein 1 (*TEP1*) in a meaningful way. Likewise, the control gene, *RPS17*, was not differentially regulated by the primary treatment. Therefore, a bacterial infection early in life increases the amount of *NOS*—and perhaps *PPO6*—mRNA when a mosquito encounters another bacterial infection later in life.

### 3.5. Injury and Infection Early in Life Improves the Survival of Mosquitoes that Become Infected with Bacteria Later in Life

**Figure 6:**
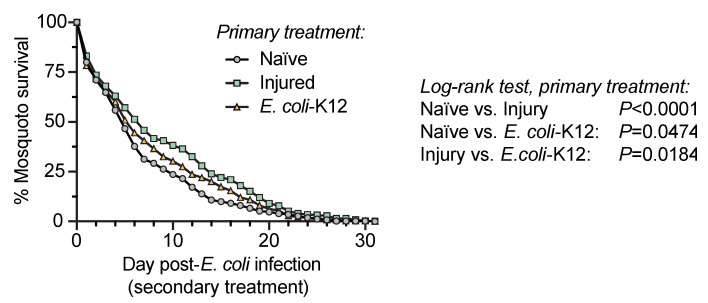
Mosquito survival after the secondary treatment. Five days after the primary treatment of naïve, injury, or infection with *E. coli*-K12, all the mosquitoes were infected with *E. coli*-GFP and their survival was tracked every day until all the mosquitoes had died.

Because a mosquito that experiences an infection early in life is better able to kill bacteria acquired later in life, we asked whether this heightened immune response translates into an increased probability that the mosquito survives a secondary infection (Figure 6). The answer to this question is complex. When mosquitoes were subjected to one of the three primary treatments on day 3 and were then infected with *E. coli*-GFP on day 8, the mosquitoes that received the injury primary treatment had the highest survival. The mosquitoes that received the *E. coli*-K12 primary treatment displayed intermediate survival, and the mosquitoes that received the naïve primary treatment experienced the lowest survival. However, these differences were modest. Specifically, all three groups experienced rapid mortality over the first 3 days following *E. coli*-GFP infection, with the slopes of the survival curves (m) being similar (−10.6, −10.9 and −11.4). Over the next four days, the mosquitoes from the naïve group died faster (m = −9.1) than the mosquitoes from the *E. coli*-K12 (m = −7.3) and injured (m = −5.6) groups, but by the 23rd day after *E. coli*-GFP infection—when the adults were 31 days old—the survival in all three groups was less than 5%. Therefore, we conclude that injury early in life, and to a lesser extent an *E. coli*-K12 infection, prepares mosquitoes to better survive a bacterial infection acquired later in life.

## 4. Discussion

Although significant progress has been made toward understanding how a prior immune challenge safeguards a mosquito from a subsequent infection, the type of challenge a mosquito experiences impacts the probability, specificity, and strength of protection. The present study investigated how mosquitoes infected with *E. coli* respond to a second infection with *E. coli*, and the findings highlight how the protection conferred by the initial infection is associated with changes in hemocyte density, hemocyte activity, and immune gene expression.

Our initial experiments showed that an infection with *E. coli* enhances the ability of mosquitoes to kill the same bacterium when it is reencountered later in life. However, hemocoelic bacterial infections acquired by adults persist for weeks and are rarely cleared [39,40]. Therefore, the protection observed in this study occurs during a co-infection with the same bacterial species, or a homologous infection. We did not address whether protection extends to other types of infections, or heterologous infections. In other insect species, the protection from reinfection is sometimes specific to a homologous infection but sometimes extends to a heterologous infection [7,9,11,15,28,41,42]. Regardless, the protection observed here likely occurs because of heightened immune awareness due to an already existing infection instead of memory arising from an earlier, cured infection. For that reason, we refrain from referring to our observations as immune memory because this would have required that the second infection be challenging a quiescent immune system that had been primed in the past [6].

Hemocytes—functionally divided into granulocytes, oenocytoids and promehocytes—are the primary immune cells of mosquitoes [43]. These cells phagocytose pathogens, drive the melanization response, and produce humoral immune factors. In the present study, we uncovered that mosquitoes that receive two infections have more hemocytes available to quell the infection than mosquitoes that experience a single infection. Studies on *E. coli*-infected adult *A. gambiae* have documented an infection-induced increase in the number of hemocytes [35,37,44,45], and this increase extends to when mosquitoes acquire the *E. coli* infection during the larval stage and hemocytes are counted in adults [46]. Therefore, we predict that the surge in hemocytes associated with the initial infection—by the mitosis of circulating hemocytes [45]—carries over to strengthen the fight against the second infection. Moreover, investigators employing a very different technique reported that when mosquitoes are first infected with malaria, cured, and subsequently reinfected with malaria, the protection from the second infection is driven by hemocyte differentiation [20,47,48]. Collectively, these data suggest that surviving an infection may trigger a protective response that is advantageous when a mosquito faces a second infection. This is supported by the finding that blood feeding by itself induces an immediate increase in the number and activation of hemocytes [44,49,50,51,52], which prepares the mosquito for the possibility of encountering a blood-borne pathogen. The fact that 20-hydroxyecdysone (20E)—a hormone produced in response to blood feeding—induces hemocyte activation and other immune processes supports that hypothesis [53,54]. The protective effect of hemocytes transcends mosquitoes; hemocytes also defend the fruit fly, *Drosophila melanogaster*; the mealworm beetle, *Tenebrio molitor*; and the greater wax moth, *Galleria mellonella*, from reinfection [7,9,55].

Although two infections increased the number of hemocytes, two infections also decreased the phagocytic activity of individual hemocytes against the second infection. We found a similar inverse correlation when studying cellular immunity in larvae and differently-aged adults; larvae have more hemocytes than adults but a smaller percentage of hemocytes engage in phagocytosis [37]. This contrasts with mosquitoes that were infected with *E. coli* as larvae and reinfected with *E. coli* as adults, where the number of hemocytes in mosquitoes infected twice was higher and so was their phagocytic activity [46]. This latter finding is similar to the strengthened phagocytosis response observed in mosquitoes treated with 20E [53]. Although the findings we present here may seem counterintuitive, we hypothesize that in mosquitoes infected twice, the activity of individual hemocytes is lower because more hemocytes are available to quell the infection and other immune processes are likely also strengthened, therefore reducing the phagocytic burden of each individual hemocyte. This is supported by our finding that mosquitoes infected twice are more capable of killing bacteria in their hemocoel than mosquitoes infected once. An alternate explanation, however, is that the phagocytosis of bacteria from the initial infection that has not been fully cleared reduces the capacity of hemocytes to phagocytose bacteria from the second infection. Regardless, experiments in the silkworm, *Bombyx mori,* and *D. melanogaster* have demonstrated that phagocytosis is key to overcoming a second infection; for example, blocking phagocytosis in fruit flies eliminates the priming of the immune response [9,42].

In addition to an increase in hemocytes, mosquitoes infected twice expressed nitric oxide synthase (*NOS*)—and perhaps prophenoloxidase 6 (*PPO6*)—more highly than mosquitoes that had been infected only once. Elevated expression of immune genes is associated with immune priming in mosquitoes [17,27,28,46,47], as well as in beetles and moths [56,57,58]. Particularly interesting is our finding that *NOS* is expressed more highly in mosquitoes infected twice. NOS is produced by hemocytes and is a major contributor to the antibacterial response in the hemocoel [59]. Moreover, NOS-produced nitric oxide modulates circulatory physiology in *A. gambiae*; *D. melanogaster*; the Vietnamese stick insects, *Baculum extradentatum*; and the migratory locust, *Locusta migratoria* [60,61,62,63], which likely impacts the bacteria-hemocyte interplay because the circulatory and immune systems are functionally integrated [40,64,65]. Another interesting observation was the observed trend of increased *PPO6* expression in mosquitoes infected twice. Phenoloxidases drive the melanization pathway [66,67], and melanization potential is elevated in some primed insects, although this is not a universal feature across Insecta [10,16,42,58].

When mosquito survival was tracked beginning with the secondary treatment, mosquitoes that had been infected twice had a higher survivorship than mosquitoes that had received the naïve primary treatment. However, the highest degree of survival was experienced by mosquitoes that had been injured earlier in life. Enhanced survival after immune stimulation has also been observed in *A. aegypti* [28], but one difference between those findings and ours is that we observed that an injury primary treatment resulted in the highest survival whereas in *A. aegypti* the highest survival was in mosquitoes that had been infected twice. Similarly, increased survival following a hemocoelic priming infection also occurs in other insects, such as *D. melanogaster*; *T. molitor*; the red flour beetle, *Tribolium castaneum*; and the buff-tailed bumblebee, *Bombus terrestris* [7,9,11,41].

The experiments presented herein chronicle the effects of a hemocoelic bacterial infection acquired early in adulthood on another infection acquired later in adulthood. However, mosquitoes are holometabolous insects that undergo complex transformations during their development. Because the aquatic environment of larvae is rife with bacteria, infections often begin before metamorphosis. Bacterial infections in the larval hemocoel of *A. gambiae* are transstadially passaged to adults and result in developmental and longevity costs [68]. However, infected larvae that survive to adulthood are better equipped to kill bacteria in their hemocoel, and do so with an enhanced number of hemocytes and increased expression of immune genes [46]. An analogous phenomenon occurs in *A. aegypti*, where exposing larvae to bacteria or inactivated dengue virus produces adults with enhanced immune gene expression, heightened immune activity, and increased resistance to dengue [69,70,71]. Likewise, *A. aegypti* and *Culex quinquefasciatus* larvae that are infected with *Bacillus* sp. eclose into adults that are more resistant to filarial nematodes and malaria parasites [72,73,74]. Therefore, the protection effects we observed are not restricted to immune challenges that occur during adulthood.

Beyond the experiments presented herein, several questions remain. First, this study focused on a homologous infection: a primary treatment with *E. coli* was followed by a secondary treatment with *E. coli*. Whether similar phenotypes are observed following heterologous infections—and in different combinations of primary and secondary treatments—should be investigated. Second, this study investigated the biology of circulating hemocytes and did not assess the contribution of sessile hemocytes. In mosquitoes, approximately 75% of hemocytes circulate with the hemolymph and 25% of hemocytes are sessile [45]. These sessile hemocytes adhere to many different tissues, and a specific population, called periostial hemocytes, adheres to the heart and actively phagocytoses pathogens as they flow toward the mosquito’s major circulatory organ [40,45,64,75]. Sessile hemocytes can become circulating and vice versa, and in *D. melanogaster* sessile hemocytes also function as a hematopoietic source of circulating hemocytes [64,76,77,78,79]. Whether an infection alters how sessile hemocytes respond to a second infection remains unknown. Finally, whether the perturbation of hemocyte function or nitric oxide activity alters the priming phenotype is a question for future study.

## 5. Conclusions

This study adds to the growing body of evidence showing that infection has long-lasting effects on the immunological prowess of an insect. This study also highlights the dynamic nature of the mosquito immune response and how one infection prepares a mosquito to survive a subsequent infection.

## Figures and Tables

**Table 1:** Genes assayed by Real-Time PCR (RT-PCR), including gene IDs and primer sequences.

Gene	VectorBase ID ^1^	Primer sequences: 5’→3’		Amplicon (bp) ^2,3^	
		Forward	Reverse	Transcript	Genomic
*RPS7*	AGAP010592	GACGGATCCCAGCTGATAAA	GTTCTCTGGGAATTCGAACG	132	281
*RPS17*	AGAP004887	GACGAAACCACTGCGTAACA	TGCTCCAGTGCTGAAACATC	153	264
*PPO6*	AGAP004977	AGAGCCACTACCGGAAGGAT	TCGATGCTCTCAGCAATACG	174	242
*NOS*	AGAP029502	CAAGAGTGGGACCACATCAA	ACCCTTCTGGACCATCTCCT	129	210
*LYSC1*	AGAP007347	ACGGCATCTTCCAGATCAAC	CATTGCAGTGGTTCTTCCAG	180	259
*CEC1*	AGAP000693	GCTGAAGAAGCTGGGAAAGA	ATGTTAGCAGAGCCGTCGTC	158	247
*TEP1*	AGAP010815	GACGTCCAAATACGGATCTCA	CTTTCAGGCATCACCCGTAT	184	NA

^1^ IDs are from the AgamP4 assembly at www.vectorbase.org. ^2^ Amplicon sizes are based on the sequences in Vectorbase. ^3^ “NA”: a primer spans an exon-intron junction, and thus genomic DNA does not amplify.

## References

[1] Delves P.J., Martin S.J., Burton D.R., Roitt I.M. (2011). Roitt’s Essential Immunology.

[2] Masri L., Cremer S. (2014). Individual and social immunisation in insects. Trends Immunol..

[3] Shaw D.K., Tate A.T., Schneider D.S., Levashina E.A., Kagan J.C., Pal U., Fikrig E., Pedra J.H.F. (2018). Vector immunity and evolutionary ecology: The harmonious dissonance. Trends Immunol..

[4] Sheehan G., Farrell G., Kavanagh K. (2020). Immune priming: The secret weapon of the insect world. Virulence.

[5] Cooper D., Eleftherianos I. (2017). Memory and specificity in the insect immune system: Current perspectives and future challenges. Front. Immunol..

[6] Melillo D., Marino R., Italiani P., Boraschi D. (2018). Innate immune memory in invertebrate metazoans: A critical appraisal. Front. Immunol..

[7] Dhinaut J., Chogne M., Moret Y. (2018). Immune priming specificity within and across generations reveals the range of pathogens affecting evolution of immunity in an insect. J. Anim. Ecol..

[8] Ferro K., Peuss R., Yang W., Rosenstiel P., Schulenburg H., Kurtz J. (2019). Experimental evolution of immunological specificity. Proc. Natl. Acad. Sci. USA.

[9] Pham L.N., Dionne M.S., Shirasu-Hiza M., Schneider D.S. (2007). A specific primed immune response in *Drosophila* is dependent on phagocytes. PLoS Pathog..

[10] Moret Y., Siva-Jothy M.T. (2003). Adaptive innate immunity? Responsive-mode prophylaxis in the mealworm beetle, *Tenebrio molitor*. Proc. Biol. Sci..

[11] Sadd B.M., Schmid-Hempel P. (2006). Insect immunity shows specificity in protection upon secondary pathogen exposure. Curr. Biol..

[12] Gonzalez-Tokman D.M., Gonzalez-Santoyo I., Lanz-Mendoza H., Aguilar A.C. (2010). Territorial damselflies do not show immunological priming in the wild. Physiol. Entomol..

[13] Reber A., Chapuisat M. (2012). No evidence for immune priming in ants exposed to a fungal pathogen. PLoS ONE.

[14] Patrnogic J., Castillo J.C., Shokal U., Yadav S., Kenney E., Heryanto C., Ozakman Y., Eleftherianos I. (2018). Pre-exposure to non-pathogenic bacteria does not protect *Drosophila* against the entomopathogenic bacterium *Photorhabdus*. PLoS ONE.

[15] Futo M., Sell M.P., Kutzer M.A.M., Kurtz J. (2017). Specificity of oral immune priming in the red flour beetle *Tribolium castaneum*. Biol. Lett..

[16] Bargielowski I., Koella J.C. (2009). A possible mechanism for the suppression of *Plasmodium berghei* development in the mosquito *Anopheles gambiae* by the microsporidian *Vavraia culicis*. PLoS ONE.

[17] Contreras-Garduno J., Rodriguez M.C., Hernandez-Martinez S., Martinez-Barnetche J., Alvarado-Delgado A., Izquierdo J., Herrera-Ortiz A., Moreno-Garcia M., Velazquez-Meza M.E., Valverde V. (2015). *Plasmodium berghei* induced priming in *Anopheles albimanus* independently of bacterial co-infection. Dev. Comp. Immunol..

[18] Contreras-Garduno J., Rodriguez M.C., Rodriguez M.H., Alvarado-Delgado A., Lanz-Mendoza H. (2014). Cost of immune priming within generations: Trade-off between infection and reproduction. Microbes Infect..

[19] Lowenberger C.A., Kamal S., Chiles J., Paskewitz S., Bulet P., Hoffmann J.A., Christensen B.M. (1999). Mosquito-*Plasmodium* interactions in response to immune activation of the vector. Exp. Parasitol..

[20] Rodrigues J., Brayner F.A., Alves L.C., Dixit R., Barillas-Mury C. (2010). Hemocyte differentiation mediates innate immune memory in *Anopheles gambiae* mosquitoes. Science.

[21] Herren J.K., Mbaisi L., Mararo E., Makhulu E.E., Mobegi V.A., Butungi H., Mancini M.V., Oundo J.W., Teal E.T., Pinaud S. (2020). A microsporidian impairs *Plasmodium falciparum* transmission in *Anopheles arabiensis* mosquitoes. Nat. Commun..

[22] Kambris Z., Cook P.E., Phuc H.K., Sinkins S.P. (2009). Immune activation by life-shortening *Wolbachia* and reduced filarial competence in mosquitoes. Science.

[23] Lowenberger C.A., Ferdig M.T., Bulet P., Khalili S., Hoffmann J.A., Christensen B.M. (1996). *Aedes aegypti*: Induced antibacterial proteins reduce the establishment and development of *Brugia malayi*. Exp. Parasitol..

[24] Ye Y.H., Woolfit M., Rances E., O’Neill S.L., McGraw E.A. (2013). *Wolbachia*-associated bacterial protection in the mosquito *Aedes aegypti*. PLoS Negl. Trop. Dis..

[25] Aliota M.T., Peinado S.A., Velez I.D., Osorio J.E. (2016). The wMel strain of *Wolbachia* reduces transmission of Zika virus by *Aedes aegypti*. Sci. Rep..

[26] Serrato-Salas J., Izquierdo-Sanchez J., Arguello M., Conde R., Alvarado-Delgado A., Lanz-Mendoza H. (2018). *Aedes aegypti* antiviral adaptive response against DENV-2. Dev. Comp. Immunol..

[27] Cappelli A., Damiani C., Mancini M.V., Valzano M., Rossi P., Serrao A., Ricci I., Favia G. (2019). *Asaia* activates immune genes in mosquito eliciting an anti-*Plasmodium* response: Implications in malaria control. Front. Genet..

[28] Vargas V., Moreno-Garcia M., Duarte-Elguea E., Lanz-Mendoza H. (2016). Limited specificity in the injury and infection priming against bacteria in *Aedes aegypti* mosquitoes. Front. Microbiol..

[29] Dodson B.L., Hughes G.L., Paul O., Matacchiero A.C., Kramer L.D., Rasgon J.L. (2014). *Wolbachia* enhances West Nile virus (WNV) infection in the mosquito *Culex tarsalis*. PLoS Negl. Trop. Dis..

[30] Joshi D., Pan X., McFadden M.J., Bevins D., Liang X., Lu P., Thiem S., Xi Z. (2017). The maternally inheritable *Wolbachia* wAlbB induces refractoriness to *Plasmodium berghei* in *Anopheles stephensi*. Front. Microbiol..

[31] Hughes G.L., Koga R., Xue P., Fukatsu T., Rasgon J.L. (2011). *Wolbachia* infections are virulent and inhibit the human malaria parasite *Plasmodium falciparum* in *Anopheles gambiae*. PLoS Pathog..

[32] Murdock C.C., Blanford S., Hughes G.L., Rasgon J.L., Thomas M.B. (2014). Temperature alters *Plasmodium* blocking by *Wolbachia*. Sci. Rep..

[33] Zele F., Nicot A., Berthomieu A., Weill M., Duron O., Rivero A. (2014). *Wolbachia* increases susceptibility to *Plasmodium* infection in a natural system. Proc. Biol. Sci..

[34] Estevez-Lao T.Y., Boyce D.S., Honegger H.W., Hillyer J.F. (2013). Cardioacceleratory function of the neurohormone CCAP in the mosquito *Anopheles gambiae*. J. Exp. Biol..

[35] Coggins S.A., Estevez-Lao T.Y., Hillyer J.F. (2012). Increased survivorship following bacterial infection by the mosquito *Aedes aegypti* as compared to *Anopheles gambiae* correlates with increased transcriptional induction of antimicrobial peptides. Dev. Comp. Immunol..

[36] Hillyer J.F., Schmidt S.L., Fuchs J.F., Boyle J.P., Christensen B.M. (2005). Age-associated mortality in immune challenged mosquitoes (*Aedes aegypti*) correlates with a decrease in haemocyte numbers. Cell. Microbiol..

[37] League G.P., Estevez-Lao T.Y., Yan Y., Garcia-Lopez V.A., Hillyer J.F. (2017). *Anopheles gambiae* larvae mount stronger immune responses against bacterial infection than adults: Evidence of adaptive decoupling in mosquitoes. Parasit Vectors.

[38] Livak K.J., Schmittgen T.D. (2001). Analysis of relative gene expression data using real-time quantitative PCR and the 2(-Delta Delta C(T)) method. Methods.

[39] Gorman M.J., Paskewitz S.M. (2000). Persistence of infection in mosquitoes injected with bacteria. J. Invertebr. Pathol..

[40] King J.G., Hillyer J.F. (2012). Infection-induced interaction between the mosquito circulatory and immune systems. PLoS Pathog..

[41] Roth O., Sadd B.M., Schmid-Hempel P., Kurtz J. (2009). Strain-specific priming of resistance in the red flour beetle, *Tribolium castaneum*. Proc. Biol. Sci..

[42] Wu G., Li M., Liu Y., Ding Y., Yi Y. (2015). The specificity of immune priming in silkworm, *Bombyx mori*, is mediated by the phagocytic ability of granular cells. J. Insect Physiol..

[43] Hillyer J.F., Strand M.R. (2014). Mosquito hemocyte-mediated immune responses. Curr. Opin. Insect Sci..

[44] Baton L.A., Robertson A., Warr E., Strand M.R., Dimopoulos G. (2009). Genome-wide transcriptomic profiling of *Anopheles gambiae* hemocytes reveals pathogen-specific signatures upon bacterial challenge and *Plasmodium berghei* infection. BMC Genom..

[45] King J.G., Hillyer J.F. (2013). Spatial and temporal in vivo analysis of circulating and sessile immune cells in mosquitoes: Hemocyte mitosis following infection. BMC Biol..

[46] Brown L.D., Shapiro L.L.M., Thompson G.A., Estevez-Lao T.Y., Hillyer J.F. (2019). Transstadial immune activation in a mosquito: Adults that emerge from infected larvae have stronger antibacterial activity in their hemocoel yet increased susceptibility to malaria infection. Ecol. Evol..

[47] Ramirez J.L., de Almeida Oliveira G., Calvo E., Dalli J., Colas R.A., Serhan C.N., Ribeiro J.M., Barillas-Mury C. (2015). A mosquito lipoxin/lipocalin complex mediates innate immune priming in *Anopheles gambiae*. Nat. Commun..

[48] Ramirez J.L., Garver L.S., Brayner F.A., Alves L.C., Rodrigues J., Molina-Cruz A., Barillas-Mury C. (2014). The role of hemocytes in *Anopheles gambiae* antiplasmodial immunity. J Innate Immun..

[49] Smith R.C., King J.G., Tao D., Zeleznik O.A., Brando C., Thallinger G.G., Dinglasan R.R. (2016). Molecular profiling of phagocytic immune cells in *Anopheles gambiae* reveals integral roles for hemocytes in mosquito innate immunity. Mol. Cell. Proteom..

[50] Bryant W.B., Michel K. (2014). Blood feeding induces hemocyte proliferation and activation in the African malaria mosquito, *Anopheles gambiae* Giles. J. Exp. Biol..

[51] Bryant W.B., Michel K. (2016). *Anopheles gambiae* hemocytes exhibit transient states of activation. Dev. Comp. Immunol..

[52] Castillo J., Brown M.R., Strand M.R. (2011). Blood feeding and insulin-like peptide 3 stimulate proliferation of hemocytes in the mosquito *Aedes aegypti*. PLoS Pathog..

[53] Reynolds R.A., Kwon H., Smith R.C. (2020). 20-Hydroxyecdysone primes innate immune responses that limit bacterial and malarial parasite survival in *Anopheles gambiae*. mSphere.

[54] Upton L.M., Povelones M., Christophides G.K. (2015). *Anopheles gambiae* blood feeding initiates an anticipatory defense response to *Plasmodium berghei*. J. Innate Immun..

[55] Fallon J.P., Troy N., Kavanagh K. (2011). Pre-exposure of *Galleria mellonella* larvae to different doses of *Aspergillus fumigatus* conidia causes differential activation of cellular and humoral immune responses. Virulence.

[56] Greenwood J.M., Milutinovic B., Peuss R., Behrens S., Esser D., Rosenstiel P., Schulenburg H., Kurtz J. (2017). Oral immune priming with *Bacillus thuringiensis* induces a shift in the gene expression of *Tribolium castaneum* larvae. BMC Genom..

[57] Bergin D., Murphy L., Keenan J., Clynes M., Kavanagh K. (2006). Pre-exposure to yeast protects larvae of *Galleria mellonella* from a subsequent lethal infection by *Candida albicans* and is mediated by the increased expression of antimicrobial peptides. Microbes Infect..

[58] Vertyporokh L., Wojda I. (2020). Immune response of *Galleria mellonella* after injection with non-lethal and lethal dosages of *Candida albicans*. J. Invertebr. Pathol..

[59] Hillyer J.F., Estevez-Lao T.Y. (2010). Nitric oxide is an essential component of the hemocyte-mediated mosquito immune response against bacteria. Dev. Comp. Immunol..

[60] Estevez-Lao T.Y., Sigle L.T., Gomez S.N., Hillyer J.F. (2020). Nitric oxide produced by periostial hemocytes modulates the bacterial infection induced reduction of the mosquito heart rate. J. Exp. Biol..

[61] Broderick K.E., Feala J., McCulloch A., Paternostro G., Sharma V.S., Pilz R.B., Boss G.R. (2006). The nitric oxide scavenger cobinamide profoundly improves survival in a *Drosophila melanogaster* model of bacterial sepsis. FASEB J..

[62] Bullerjahn A., Mentel T., Pfluger H.J., Stevenson P.A. (2006). Nitric oxide: A co-modulator of efferent peptidergic neurosecretory cells including a unique octopaminergic neurone innervating locust heart. Cell Tissue Res..

[63] da Silva R., da Silva S.R., Lange A.B. (2012). The regulation of cardiac activity by nitric oxide (NO) in the Vietnamese stick insect, *Baculum extradentatum*. Cell. Signal..

[64] Sigle L.T., Hillyer J.F. (2016). Mosquito hemocytes preferentially aggregate and phagocytose pathogens in the periostial regions of the heart that experience the most hemolymph flow. Dev. Comp. Immunol..

[65] Hillyer J.F. (2015). Integrated immune and cardiovascular function in Pancrustacea: Lessons from the insects. Integr. Comp. Biol..

[66] Hillyer J.F. (2016). Insect immunology and hematopoiesis. Dev. Comp. Immunol..

[67] Whitten M.M.A., Coates C.J. (2017). Re-evaluation of insect melanogenesis research: Views from the dark side. Pigment Cell Melanoma Res..

[68] Brown L.D., Thompson G.A., Hillyer J.F. (2018). Transstadial transmission of larval hemocoelic infection negatively affects development and adult female longevity in the mosquito *Anopheles gambiae*. J. Invertebr. Pathol..

[69] Carlson J.S., Short S.M., Anglero-Rodriguez Y.I., Dimopoulos G. (2020). Larval exposure to bacteria modulates arbovirus infection and immune gene expression in adult *Aedes aegypti*. Dev. Comp. Immunol..

[70] Vargas V., Cime-Castillo J., Lanz-Mendoza H. (2020). Immune priming with inactive dengue virus during the larval stage of *Aedes aegypti* protects against the infection in adult mosquitoes. Sci. Rep..

[71] Moreno-Garcia M., Vargas V., Ramirez-Bello I., Hernandez-Martinez G., Lanz-Mendoza H. (2015). Bacterial exposure at the larval stage induced sexual immune dimorphism and priming in adult *Aedes aegypti* mosquitoes. PLoS ONE.

[72] Kala M.K., Gunasekaran K. (1999). Effect of *Bacillus thuringiensis* ssp. israelensis on the development of *Plasmodium gallinaceum* in *Aedes aegypti* (Diptera: Culicidae). Ann. Trop. Med. Parasitol..

[73] Mahapatra N., Hazra R.K., Rup S., Acharya A.S., Dash A.P. (1999). *Bacillus sphaericus* interferes with the development of *Brugia malayi* in *Aedes aegypti*. J. Helminthol..

[74] Paily K.P., Geetha I., Kumar B.A., Balaraman K. (2012). *Bacillus sphaericus* in the adults of *Culex quinquefasciatus* mosquitoes emerged from treated larvae and its effect on development of the filarial parasite, *Wuchereria bancrofti*. Parasitol. Res..

[75] League G.P., Hillyer J.F. (2016). Functional integration of the circulatory, immune, and respiratory systems in mosquito larvae: Pathogen killing in the hemocyte-rich tracheal tufts. BMC Biol..

[76] Babcock D.T., Brock A.R., Fish G.S., Wang Y., Perrin L., Krasnow M.A., Galko M.J. (2008). Circulating blood cells function as a surveillance system for damaged tissue in *Drosophila* larvae. Proc. Natl. Acad. Sci. USA.

[77] Markus R., Laurinyecz B., Kurucz E., Honti V., Bajusz I., Sipos B., Somogyi K., Kronhamn J., Hultmark D., Ando I. (2009). Sessile hemocytes as a hematopoietic compartment in *Drosophila melanogaster*. Proc. Natl. Acad. Sci. USA.

[78] Sigle L.T., Hillyer J.F. (2018). Mosquito hemocytes associate with circulatory structures that support intracardiac retrograde hemolymph flow. Front. Physiol..

[79] Williams M.J., Wiklund M.L., Wikman S., Hultmark D. (2006). Rac1 signalling in the *Drosophila* larval cellular immune response. J. Cell Sci..

